# Changes in Exercise Capacity of Patients With Obstructive Sleep Apnea Following Treatment With Continuous Positive Airway Pressure

**DOI:** 10.7759/cureus.21729

**Published:** 2022-01-30

**Authors:** Konstantinos M Pigakis, Athanasios Voulgaris, Evagelia Nena, Aggeliki Kontopodi, Paschalis Steiropoulos

**Affiliations:** 1 Respiratory Medicine, Creta Interclinic Hospital, Heraklion, GRC; 2 Respiratory Medicine, Faculty of Health Sciences, Democritus University of Thrace, Alexandroupoli, GRC; 3 Public Health Sciences, Faculty of Health Sciences, Democritus University of Thrace, Alexandroupoli, GRC

**Keywords:** exercise, physical activity, continuous positive airway pressure, cardiopulmonary exercise testing, obstructive sleep apnea

## Abstract

Introduction: Patients with obstructive sleep apnea (OSA) frequently complain of fatigue during exercise. Treatment with continuous positive airway pressure (CPAP) ameliorates OSA-related symptoms and may reduce the burden of OSA on coexistent diseases. However, the role of CPAP on exercise capacity in OSA has not been fully investigated.

Aim: The aim of this study is to assess exercise capacity in a group of newly diagnosed OSA patients, without known comorbidities, following treatment with CPAP.

Methods: Consecutively diagnosed OSA patients by polysomnography completed the International Physical Activity Questionnaire (IPAQ) and underwent cardiopulmonary exercise testing (CPET) and pulmonary function testing at baseline of OSA diagnosis three months after adherence to CPAP treatment.

Results: A total of 40 OSA patients (Apnea-Hypopnea Index (ΑΗΙ)>15 events/hour) of whom 29 (72.5%) males with an average age of 42±2.5 years were enrolled in the study. OSA patients had a mean peak oxygen uptake (V̇O_2_) value of 40.3 ±8.4 ml/kg/min (77.7±15%), which was improved after three months on CPAP treatment, 47.6±7.9 ml/kg/min (92.9±10.5%). (p=0.002). In addition, patients’ mean work (W) value increased significantly from baseline to three months of treatment with CPAP (101.5±30 watts vs 78.6±18.5 watts. p=0.015, respectively). There were no significant differences in terms of physical activity, as noted in IPAQ, before and after OSA therapy (p=0.075).

Conclusions: In the present study, OSA is associated with impaired exercise capacity, which seems to be improved after short-term treatment with PAP. Further evidence is warranted to elucidate whether CPET could be routinely used to monitor treatment responses of OSA with CPAP.

## Introduction

Obstructive sleep apnea syndrome (OSAS) is characterized by repeated episodes of partial (hypopneas) or complete (apneas) upper airway obstruction during sleep, resulting in oxyhemoglobin desaturation and sleep fragmentation [1]. Currently, OSAS is the most frequent breathing disorder during sleep, with global estimates reaching up to one billion adults of middle age [2]. In addition, there is a predilection of OSAS for the male gender as well as in individuals with increasing BMI levels; being highest amongst those with morbid obesity [3].

OSAS is related to a variety of symptoms, which can be divided into those that present during wakefulness such as daytime sleepiness, morning fatigue, and headaches, and those that emerge at night like snoring, choking, gasping, and nocturia [4]. From a pathophysiological point of view, individuals with OSAS demonstrate abnormal gas exchange with oscillations of intermittent nocturnal hypoxia and hypercapnia as well as high negative intrathoracic pressure to overcome the apneic events. These could result in autonomic nerve dysfunction, increased heart afterload, and ultimately left ventricular dysfunction and hemodynamic impairment [5].

Polysomnography (PSG) is the gold standard for diagnosing OSAS, with cardiorespiratory polygraphy deemed to be an acceptable alternative [6-8]. According to the apnea-hypopnea index (AHI) indicating the number of apneic or hypopneic episodes per hour of sleep, OSAS is classified as mild, moderate, or severe [9]. Somnolence is the principal symptom in OSAS, which can be evaluated using the Epworth questionnaire. Therapy is recommended in all patients with moderate and severe OSAS (AHI > 15 events/hour), and in patients with mild OSAS with comorbidities [10]. The treatment choices consist of continuous positive airway pressure (CPAP), mandibular mechanisms, operation, and hypoglossal nerve stimulation [11]. CPAP continues to be the treatment of choice for moderate and severe OSAS. The latest evidence suggests that OSAS patients commonly exhibit increased levels of fatigue during exercise, muscle weakness, and reduced body reserves in response to high energy demands [7,12,13]. Cardiovascular comorbidities (such as heart failure and hypertension), obesity, and obesity-associated lung function abnormalities (such as reduced functional residual capacity) are highly prevalent among patients with OSAS and may further impair exercise capacity, while the lack of regular physical activity, especially of moderate and vigorous intensity, has been also suggested to interfere with the pathogenesis of OSAS.

Cardiopulmonary exercise testing (CPET) is a non-invasive technique, which provides an objective quantitative assessment of metabolic, pulmonary, cardiovascular, neuropsychological, muscular, hematopoietic, and skeletal systems responses during exercise [14]. Simply, CPET assesses the maximum aerobic capacity and is used in a wide range of medical applications for the estimation and investigation of undiagnosed exercise intolerance and the actual determination of functional capacity. It is used in patient management suggesting that resting lung and cardiac function testing cannot predict with certainly exercise performance and functional capacity [14,15]. 

Currently, the published data on the effect of continuous positive airway pressure (CPAP) treatment on exercise capacity parameters, as assessed by CPET, among OSAS patients, are conflicting [16]. Duration of CPAP application varies widely in these studies (from a few days to several weeks), making it difficult to distinguish between short- and long-term effects of CPAP application, while OSAS patients with various comorbidities have been assessed, increasing the controversy in the field. Similarly, published data regarding the effect of solely CPAP treatment on the level and intensity of physical activity in OSAS patients are currently scarce. Therefore, the aim of this study was to evaluate the impact of CPAP treatment for 12 weeks on (a) exercise capacity, as assessed by maximum CPET, and (b) physical activity, as assessed by the International Physical Activity Questionnaire (IPAQ), among patients with moderate or severe OSAS without comorbidities.

## Materials and methods

Study participants

Forty consecutive patients, newly diagnosed with moderate and severe OSAS, and without other comorbidities were included. The study protocol was approved by the ethics committee of the Creta InterClinic Hospital (Registry number: 2020/129). The study was conducted in accordance with the Helsinki Declaration of Human Rights and patients signed consent for inclusion.

Patients’ recruitment started in October 2018 and was completed in October 2019. The patients' last follow-up after baseline evaluation took place in January 2020. Inclusion criteria were the following: new diagnosis of moderate to severe OSAS without any previous treatment and patient’s agreement to participate in the study. Exclusion criteria were: exclusively central sleep apnea on PSG, prior use of CPAP, upper airway and craniofacial deformities, known medical history of cardiovascular, respiratory, orthopaedical, endocrine, metabolic, neurological, psychiatric, and haematological diseases, anaemia (haemoglobin of < 13.5 g/dl for men and < 12.5 g/dl for women), regular moderate to intense exercise (i.e. more than three times per week), and body weight > 130 kg due to technical specifications to complete CPET.

According to the study protocol, a detailed history concerning known medical conditions, current medication, and smoking habits were routinely obtained from all included individuals. In addition, a standard clinical examination was performed in all participants, which included the following: recording of anthropometric characteristics (height, weight, neck, waist and hip circumference, ΒΜΙ, blood pressure, and oxygen saturation). Moreover, all patients underwent pulmonary function testing using spirometry, cardiac evaluation with echocardiography and electrocardiogram (ECG), daytime sleepiness estimation with Epworth Sleepiness Scale (ESS), and assessment of physical activity with the IPAQ, before and after three months of CPAP therapy. IPAQ is a self-administered questionnaire that assesses overall the physical activities that people do as part of their everyday lives during the last seven days (see Appendix) [17,18]. According to the results of the Epworth questionnaire, somnolence was classified as normal (0-10 points), mild (11-12 points), moderate (13-15 points), and severe (16-24 points). According to the IPAQ, physical activity (PA) was classified as PA during walking (low intensity), PA of moderate intensity, and PA of high intensity and average metabolic equivalents (METs)/day were calculated.

Polysomnography

Unattended PSG was conducted between 10:00 pm and 06:00 am and the variables were recorded in the computer system (Nox A1® PSG system, Nox Medical, Reykjavík, Iceland). Sleep staging was based on a standard montage of electroencephalogram (EEG), electro-occulogram, electromyogram, and ECG signals. Pulse oximetry was registered, and airflow was detected with the help of oronasal thermistors for apneas and nasal pressure for hypopneas in combination. Thoracic cage and abdominal motion were detected using inductive plethysmography. Recordings were manually scored according to the standard criteria [9]. Apnea was defined as a ≥90% decrease in airflow for a minimum of 10 sec. Hypopnea was defined as a ≥30% decrease in airflow for a minimum of 10 sec in combination with oxyhaemoglobin desaturation of a minimum of 3% or arousal registered by the EEG. AHI was calculated as an average of apneas and hypopneas per hour of PSG-recorded sleep time [9]. An auto titration sleep study at the sleep laboratory with CPAP was performed to set the effective pressure for resolution of all respiratory events and normalization of the sleep architecture. OSAS patients who tolerated the sleep study under CPAP were prescribed an Auto-CPAP device (AirSense™ 10, ResMed Inc., San Diego, California) for home long-term OSAS treatment.

Cardiopulmonary exercise testing

The cardiopulmonary exercise test (CPET) is a method that allows the evaluation of body functional capacity in a non-invasive way [19-21]. The cardiopulmonary exercise test has a wide range of clinical applications that aim to evaluate the level of exercise capacity [20-23]. This testing provides data on the respiratory gas exchange, including oxygen uptake (VO2), carbon dioxide output (VCO2), tidal volume (VT), minute ventilation (VE), and other variables such as ECG, blood pressure, and oxygen saturation. The VO2 is the most important parameter during CPET [19-22].

The CPET has become an important clinical tool to evaluate exercise capacity, as well as the systems (pulmonary, cardiovascular, musculoskeletal, and haematological) involved in exercise [19,20,24,25]. Finally, CPET assesses the oxygen transport system and provides a lot of information about the physical condition of the human body. Overall, CPET is an effective way to evaluate the causes of poor exercise capacity [14].

In this study protocol, CPET was performed over a cycloergometer (Ergocard Clinical with ExpAir Software, Medisoft Group, Sorinnes, Belgium) under the supervision of a respiratory physician at the laboratory of Creta Interclinic Hospital in Heraklion, Greece. Briefly, the CPET protocol consisted of the following procedure: it started with two minutes rest period followed by one minute of warm-up pedalling against with a minimal load of 20 watts, progressively increasing by 10 watts per minute. The duration of the test lasted from 8-12 minutes. The CPET was conducted under continuous monitoring of heart rate (HR), 12-lead ECG, and pulse oxygen saturation (SpO_2_), while systolic (SAP) and diastolic (DAP) blood pressure was recorded every two minutes with a cuff sphygmomanometer. Indication for early test termination included myocardial ischemia, complex ventricular premature beats, grade-2 or grade-3 atrioventricular block, a sudden fall in blood pressure levels by more than 20 mmHg, elevated blood pressure (SBP > 220 mmHg, DBP > 120 mmHg), SpO_2_ < 80%, confusion, vertigo, and pallor [14]. The maximum level of exercise in our study was further confirmed by achieving maximum HR > 85% of the age-predicted. Functional capacity was evaluated according to VO_2_ max, using the Weber classification: Weber A (little or no impairment): > 20 ml/kg/min, Weber B (mild to moderate impairment): 16-20 ml/kg/min, Weber C (moderate to severe impairment): 10-16 ml/kg/min, and Weber D (severe impairment): < 10 ml/kg/min [14].

Follow-up

After three months of treatment with a positive pressure device (Auto-CPAP), maximum exercise capacity, using CPET, sleepiness (using the ESS), and physical activity (using IPAQ) were re-assessed in all patients. The patients who developed a major finding during the first step of study (ischemic changes on the ECG, arrhythmias during exercise test, abnormal blood pressure response during exercise test) and the patients who showed poor treatment compliance (as evidenced by the data recorded on the device), were excluded from re-evaluation. The use of CPAP for less than four hours of sleep was considered poor treatment compliance.

Statistical analysis

All statistical analysis was carried out using SPSS Statistics for Windows, Version 17.0 (Released 2008. SPSS Inc., Chicago). Continuous variables were tested for normality of distribution with the Shapiro-Wilk test and data were expressed as mean±standard deviation (SD) accordingly. The chi-squared test was used for the comparison of percentages between groups. Correlations were tested with Pearson’s correlation coefficient, while comparisons between means were studied with the paired t-test or the non-parametric Wilcoxon Rank Sum test, depending on the distribution of values. Reported p-values are two-tailed and significance was defined at p<0.05.

## Results

Overall, 40 newly diagnosed OSAS patients without other comorbidities (29 males and 11 females, mean age of 42.00±2.50) participated in the study. Male gender was prevalent in our study group, with a male to female (M/F) ratio of 2.63. Included participants had a mean baseline BMI of 37.60 ± 3.20 kg/m2, mean AHI of 37.86 ± 19.67 events/hour, while ESS score was 11.81 ± 5.32 points, baseline mean IPAQ score was 567 ± 23.85 METs. The main VO2 max prior to CPAP treatment was 77,70 ± 15,00 % (normal value is regarded as VO2 max > 80%) and in absolute values was 40,30 ± 4,40 ml/kg/min. The patients’ baseline characteristics are shown in Table 1. None of the participants demonstrated a decrease in functional capacity at baseline as stated by the Webber classification (Table 2). Moreover, patients’ pulmonary function testing (PFTs) was without abnormalities.

**Table 1:** Baseline parameters of the study before and after CPAP AHI: apnoea hypopnoea index; BMI: body mass index; CPAP: continuous positive airway pressure; DBP: diastolic blood pressure; ESS: Epworth Sleepiness Scale; F: female; FEV_1_: forced expiratory volume during the first second; FVC: forced vital capacity; HR: heart rate; M: male; ODI: oxygen desaturation index; METS: metabolic equivalent of task; PA: physical activity; SBP: systolic blood pressure; VE max: maximal ventilation; VO_2_ AT: oxygen consumption at anaerobic threshold; VO_2_ max: maximal oxygen consumption

Parameter	Before CPAP	After CPAP	p-value
Gender (M/F)	29/11 (2.63)	-	-
Age (years)	42.00 ± 2.50	-	-
BMI (kg/m^2^)	37.60 ± 3.20	37.62 ± 3.21	0.972
AHI (events/hour)	37.86 ± 19,67	-	-
ODI (desaturation Index)	35.40 ± 20.00	-	-
Average nocturnal SpO_2_	91.20 ± 3,90	-	-
Minimum nocturnal SpO_2_	72.30 ± 12.30	-	-
ESS	11.81 ± 5.32	7.5 ± 2.31	0.00001
FVC (%)	93.00 ± 15.30	99.60 ± 7.70	0.054
FEV_1_ (%)	89.70 ± 13.80	98.30 ± 8.50	0.045
FEV_1_/FVC	97.00 ± 7.70	100.00 ± 7.50	0.062
VO_2 _max (ml/kg/min)	40.30 ± 4.40	47.60 ± 7.91	0.013
% pred. VO_2_ max	77.70 ± 15.00	92.90 ± 10.50	0.002
VO_2_ AT (%)	43.70 ± 19.80	48.20 ± 14.70	0.070
VO_2_/HR (%)	114.59 ± 23.10	115.47 ± 22.00	0.090
VE max (%)	61.00 ± 9.60	59.00 ± 9.90	0.061
Resting HR	80.37 ± 13.10	72.10 ± 15.00	0.051
Resting SBP (mmHg)	129.71 ± 11.70	129.40 ± 5.80	0.061
Resting DBP (mmHg)	83.74 ± 7.50	76.83 ± 3.00	0.021
SBP max (mmHg)	145.00 ± 27.41	143.92 ± 20.10	0.070
DBP max (mmHg)	69.00 ± 12.90	84.02 ± 12.11	0.080
Exercise duration (min)	12.40 ± 2.60	13.30 ± 2.00	0.072
Walking Physical Activity (METS)	247 ± 11.42	250 ± 13.24	0.063
Moderate intensity PA (METS)	320 ± 12.43	320 ± 14.43	0.075
Hight intensity PA (METS)	0	0	-
Total PA	567 ± 23.85	570 ± 27.67	0.068

**Table 2:** Weber classification of functional limitation VO_2_: oxygen consumption

Class	Peak VO_2 _(ml/min/kg)	Severity of functional impairment
A	>20	Little or no impairment
B	16 – 20	Mild to moderate impairment
C	10 – 16	Moderate to severe impairment
D	<10	Severe limitation

After three months of CPAP treatment, the study group improved exercise capacity (VO_2_: 40.30 ± 4.40 ml/kg/ml or 77.70 ± 15.00 % of predicted to 47.60 ± 7.91 ml/kg/min or 92.90% ± 10.50 %, p = 0.013 or 0.002, respectively), resting diastolic blood pressure (DBP rest: 83.74 ± 7.50 to 76.83 ± 3.00 mmHg, p = 0.021), daytime sleepiness (ESS: 11.81 ± 5.32 to 7.15 ± 2.31 points, p = 0.00001) and the FEV_1_ (89.70% ± 13.80 to 98.30% ± 8.50, p = 0.045). To see the correlation among the OSAS severity and pulmonary function a further test was performed. Patients with severe OSAS (AHI > 30 events/hour) had lower values of respiratory function than patients with moderate OSAS (30 < AHI > 15 events/hour), but the differences were not statistically significant (Table 3). None of our patients demonstrated a decrease in functional capacity as stated by the Webber classification. Regarding exercise habits none of the patients exercised regularly (i.e., more than three times per week). The anthropometric parameters (BMI) and the level of physical activity (IPAQ score) did not change after CPAP treatment. 

**Table 3:** Baseline pulmonary function test in moderate and severe OSAS OSAS: obstructive sleep apnoea syndrome; FVC: forced vital capacity; FEV_1_: forced expiratory volume in the first second

Parameters	Moderate OSA	Severe OSA	p
Mean FVC (%)	94.33 ± 16.82	91.50 ± 13.71	0.791
Mean FEV_1 _(%)	92.67 ± 16.13	85.91 ± 9.52	0.311
FEV_1_/FVC	97.91 ± 8.22	95.82 ± 7.13	0.071

## Discussion

Ιn the present study, assessing the effects of CPAP on exercise capacity in patients with newly diagnosed moderate and severe OSAS without comorbidities, we demonstrated the following important findings: First, significant improvement in patients’ VO_2_ was observed after short-term treatment with CPAP. Second, this study demonstrated improvements in patients’ diastolic blood pressure. Finally, there was significant improvement in patients' respiratory function during wakefulness as assessed by the FEV_1_.

Various possible physiological mechanisms have been proposed to explain impaired exercise capacity in OSAS patients. A few studies have reported this fact and it has been assumed that the impairment could be due to downregulated beta-adrenergic receptors accompanying sympathetic hyperactivity [15,26-29]. Other possible mechanisms include a delay in lactate elimination. This was observed in OSAS patients during exercise and may suggest impaired glycolytic and oxidative metabolism [15,29]. Exercise impairment in OSAS patients could be explained by the existence of energetic mitochondrial disturbances [15,29]. OSAS patients may exhibit excessive daytime sleepiness, which can affect exercise capacity. Various studies have demonstrated a reduction in exercise performance under conditions of sleep deprivation [29-31].

Male gender was prevalent in our study group, with an M/F ratio of 2.63. This fact is in full agreement with previous reports in the literature [26]. Female gender hormones prevent upper airway collapse during sleep [27]. Additionally, the allocation of adipose tissue among the two genders, and the higher pharyngeal resistance among men, explain why OSAS is more common among the male population [26.28].

Evaluating the effect of CPAP treatment can provide useful information about the impact of OSAS on exercise capacity. Many studies have investigated this issue but they provide contradictory results. Although an improvement in VO_2_ has been seen after varying therapy durations ranging from one week to eight months in some studies [30-33], other studies have not reported improved VO_2_ after one to three months of CPAP treatment [34-40]. These inconsistencies may be due to differences in treatment compliance, disease severity, or physical activity levels. In one of the investigations, a significant correlation was found among AHI reduction and increase in VO_2_ after CPAP treatment [32]. In another study, two months of CPAP treatment was shown to increase VO_2_ [41]. In this study, the investigators ensured that the patients enrolled in the study did not change BMI or lifestyle during the study [41]. The high heterogeneity observed in the results of the various studies can be explained by several factors including patients’ characteristics, differences between the procedures, and criteria for determining VO_2_ {42}. An additional question concerning gender differences is whether the OSAS affect VO_2_ differently in males versus females. There is no study until today, which has assessed females only. Another potential source of heterogeneity across studies was the physical activity levels of patients and one cannot exclude the possibility that differences in physical activity levels between OSAS patients may explain, at least, the differences in VO_2_.

Previous studies reported that OSAS patients have elevated diastolic blood pressure (DBP) compared to controls [30-31]. In our research, the resting DBP, before CPAP treatment, was in the normal range, but after CPAP treatment was found to be still lower and that difference was statistically significant (p = 0.02). There was no significant difference in resting systolic blood pressure (SBP rest) among measurements prior to and after CPAP.

Previous studies described variations in spirometry in OSAS patients and noted significant associations between AHI, FEV_1_, and FVC [29,35]. In our study, the values of FVC, FEV_1_, and FEV_1_/FVC were found in normal ranges. An increase was observed in all parameters of pulmonary function, but a significant difference occurred in the mean FEV_1_ value (p = 0.045). To see the correlation between OSAS severity and pulmonary function, a further test was performed. Patients with severe OSAS (AHI > 30 events/hour) had lower values of pulmonary function than patients with moderate OSAS (30 < AHI > 15 events/hour), but the differences were not statistically significant.

Ιn this study, the mean ESS was improved and that improvement was statistically significant (p = 0.00001). Evolving evidence suggests that daytime sleepiness does not improve in mild OSAS, even with maximal compliance with CPAP [36]. Another study reported that the CPAP treatment improves sleepiness only in patients with severe OSA [37]. 

In our study, the physical activity before CPAP treatment was found to be lower than after the CPAP treatment, but the differences were not statistically significant (Table 1). Literature reported that CPAP treatment had a progressive incremental improvement in patients’ physical activity [38]. Other studies reported that CPAP treatment did not increase physical activity in the whole group of patients [39].

Certainly, our research has several limitations. First, our data were obtained from middle-aged adults, thus caution is needed in extrapolating the results to older subjects with OSAS. Secondly, the results were not analysed based on the severity of OSAS. Additionally, the number of included female patients was relatively small and thus the study results should be interpreted with caution. Moreover, the follow-up with CPAP therapy was only at three months and it is unknown whether CPAP could continue to exert its beneficial effects on a rather long-term duration (e.g., after one year).

## Conclusions

Patients with OSAS exhibit systematic detrimental effects, with an important impact on health, quality of life, and physical activity. In the present study, newly diagnosed OSAS patients without known comorbidities were evaluated. After short-term treatment with CPAP, the study group improved exercise capacity (VO_2 _max), daytime sleepiness, and respiratory function. The anthropometric measurements (BMI) and the level of physical activity did not change after CPAP treatment. Additional research is warranted to confirm the effects of CPAP treatment on exercise capacity, BMI, and physical activity in OSAS patients. 

## Appendices

International physical activity questionnaire (IPAQ)

We are interested in finding out about the kinds of physical activities (PA) that people do as part of their everyday lives.  The questions will ask you about the time you spent being physically active in the last seven days.  Please answer each question even if you do not consider yourself to be an active person.  Please think about the (PA) you do at work, as part of your house and yard work, to get from place to place, and in your spare time for recreation, exercise, or sport.

Think about all the vigorous that you did in the last seven days.  Vigorous PA refers to activities that take hard physical effort and make you breathe much harder than normal.  Think only about those PAs that you did for at least 10 minutes at a time.

1) During the last seven days, on how many days did you do vigorous PA like heavy lifting, digging, aerobics, or fast bicycling? 

_____ days per week

No PA   → Skip to question 3

2) How much time did you usually spend doing vigorous PA on one of those days?

_____ hours per day

_____ minutes per day

_____Don’t know/Not sure 

Think about all the moderate PA that you did in the last seven days.  Moderate PA refers to activities that take moderate physical effort and make you breathe somewhat harder than normal.  Think only about those PAs that you did for at least 10 minutes at a time.

3) During the last seven days, on how many days did you do moderate PA like carrying light loads, bicycling at a regular pace, or doubles tennis?  Do not include walking.

_____ days per week

No moderate physical activities    -->   Skip to question 5

4) How much time did you usually spend doing moderate PA on one of those days?

_____ hours per day

_____ minutes per day

_____Don’t know/Not sure 

Think about the time you spent walking in the last seven days.  This includes at work and at home, walking to travel from place to place, and any other walking that you have done solely for recreation, sport, exercise, or leisure.

5) During the last seven days, on how many days did you walk for at least 10 minutes at a time?  

_____ days per week

No walking  --> Skip to question 7

6) How much time did you usually spend walking on one of those days?

_____ hours per day

_____ minutes per day

_____Don’t know/Not sure 

The last question is about the time you spent sitting on weekdays during the last seven days.  Include time spent at work, at home, while doing course work, and during leisure time.  This may include time spent sitting at a desk, visiting friends, reading, or sitting or lying down to watch television.

7) During the last seven days, how much time did you spend sitting on a weekday?

_____ hours per day

_____ minutes per day

_____Don’t know/Not sure 

IPAQ scoring guidelines and physical activity classification criteria 

Α.* Physical Activity (PA) Scoring*

1) Walking PA     = 3.3 ΜΕΤs x (days with walking) x (daily minutes of walking)

2) Moderate PA  = 4 METs x (days with moderate PA) x (daily minutes of moderate PA)

3) Vigorous PA   = 8 METs x (days with vigorous PA) x (daily minutes of vigorous PA)

4) Total PA          = Walking PA + Moderate PA + Vigorous PA 

Β. *Physical Activity (PA) Classification Criteria *

1) Low PA           = Total PA < 600 METs/min/wk^-1^

2) Moderate PA = Vigorous PA > 480 or Total PA > 600 ΜΕΤs/min/wk^-1^

3) High PA          = Vigorous PA > 1500 or Total PA > 3000 ΜΕΤs/min/wk^-1^

(Notes: METs: Metabolic Equivalent; METs/min/wl^-1^: Metabolic Equivalent/Minute/Week)
